# Comparative assessment of three brushing techniques among in India

**DOI:** 10.6026/973206300220237

**Published:** 2026-01-31

**Authors:** Korukonda Vijaya Sridevi, Satyam Martha, Punithavathy R, Sri Ramya M, Kondapalli Haritha, Veena Balakrishnan, Bala Prasanthi Borusu

**Affiliations:** 1 Department of Pediatric and Preventive Dentistry, Lenora Institute of Dental Sciences, Rajamahendravaram, Andhra Pradesh, India

**Keywords:** Brushing techniques, plaque scores, Silness and Loe index

## Abstract

Dental plaque control in young children remains a challenge and the most effective brushing technique for this age group is still unclear. Therefore, it is of interest to evaluate the horizontal scrub, Fones and modified Bass methods in 120 government school children aged 6-9 years, who were randomly assigned to groups and taught their respective techniques using a cast model. Plaque levels were recorded before and after instruction using the Silness and Löe index and analyzed with ANOVA and post-hoc tests. All three methods significantly reduced plaque levels, but the modified Bass technique produced the greatest improvement. The modified Bass method achieves significantly greater plaque reduction than Fones and horizontal scrub techniques and thereby supporting evidence based recommendations for school based oral health instruction in this critical mixed dentition period.

## Background:

Oral health is one of the important aspects of health and impacts nutrition, confidence and quality of life [1]. Chewing and swallowing also means that one is well fed and lack of oral health can affect speech, social interaction and self-esteem [2]. Dental caries and periodontal diseases are very common in India both in children and adults [3]. The age category 6-9 years is a decisive developmental stage that is marked with the mixed dentition stage where there are both primary teeth as well as permanent teeth in the mouth. This is an intermediate period during which there are special difficulties in the maintenance of maximum oral hygiene owing to irregular tooth position, diverse tooth structures and the existence of partially erupted permanent dentules which are most appropriate to the deposition of plaque and consequent caries [4]. Moreover, children at this age group are becoming fine motor and manual dextrous, hence able to brush their teeth efficiently, but may be lacking the cognitive ability and the incentive to adhere to the practice of consistent oral care. Proper brushing methods at this formative stage are therefore critical to early childhood caries prevention, decreased prevalence of gingivitis and inculcation of life time oral health behaviours at this stage that will extend into adolescence and adulthood. The key etiological factor in the pathogenesis of the dental caries and gingivitis is dental plaque, a complex biofilm created by bacteria, which is fixed in an extracellular matrix. Plaque formation on the surface of teeth triggers a series of pathophysiological events such as the generation of acids that cause enamel demineralization and the development of inflammatory reactions in the gingival tissues [5]. Dental cleaning by brushing the teeth mechanically is the pillar of preventive dental care since use of chemicals is not sufficient to disorient mature biofilm structure. Nevertheless, most importantly, the success of plaque removal is greatly determined by the methods of brushing the teeth, the time and the frequency of brushing the teeth and the hand dexterity and adherence of an individual.

It has been found that the effectiveness of various types of brushing on the plaque can vary considerably; that is why it is crucial to find the most effective approaches that are particularly effective with the population of immature motor skill development. Different methods of brushing the teeth have been presented in the literature of dentistry, each differentiated by a specific stroke, orientation of the bristles and use. One of such techniques, called the Modified Bass technique that involves situating the bristles at a 45% angle relative to the gingival margin using short vibratory motions is generally recommended in adults because it is effective in cleaning both the gingival sulcus and interproximal spaces [6]. The Fones technique, which consists of large circular movements and the teeth in occlusion, is the one that is traditionally offered to children due to its simplicity and the ease of learning. Although the horizontal scrub method is generally frowned upon as it may cause abrasiveness, it is the most instinctively adopted when young children are concerned and could be of some practical benefit in the sense of understanding and doing. Although there is considerable literature on the brushing techniques, there is a dearth of comparative studies on the efficacy of the technique specifically in the 6 9 years age bracket and no consensus has been carried out concerning the most suitable method to be used during this development stage [7]. This gap in knowledge prompts the need to conduct a systematic research in order to present evidence-based suggestions on pediatric oral health education and practice. Therefore, it is of interest to compare commonly taught toothbrushing techniques (Modified Bass, Fones and horizontal scrub) in children aged 6-9 years to determine their relative effectiveness in reducing plaque and gingival inflammation during the mixed dentition stage.

## Materials and Methods:

The study was conducted among children in a government primary school, Rajanagaram, Rajahmundry andhra Pradesh, after obtaining ethical clearance from the Institutional Ethical Committee, Lenora Institute of Dental Sciences.

## Inclusion criteria:

Healthy, cooperative children aged 6-9 years without physical or mental disability who consented to participate.

## Exclusion criteria:

Children with orthodontic/prosthodontic appliances, dental caries, periodontal disease, or oral infections. A total of 120 children fulfilling the criteria were enrolled after permission from school authorities. Baseline oral hygiene was assessed using the Silness and Löe plaque index with disclosing solution applied to all teeth. Plaque was evaluated at four sites per tooth (mesiobuccal, buccal, distobuccal, palatal/lingual) and scored from 0 (no plaque) to 3 (abundant plaque). Individual scores were calculated by averaging tooth scores, followed by overall plaque index per child.

Children were randomly allocated into three groups (n=40 each):

[1] Group A: Horizontal scrub technique

[2] Group B: Fones technique

[3] Group C: Modified Bass technique

Each group was taught their respective brushing method using a cast model. Oral examinations were repeated after 15 days to record follow-up plaque scores. Data were analyzed using SPSS v26.0. Results were expressed as mean ± standard deviation.

One-way ANOVA with post hoc Tukey test compared differences between groups, while paired t-test assessed within-group changes. A p-value <0.05 was considered statistically significant and <0.01 highly significant.

## Results:

**Figure 1:**
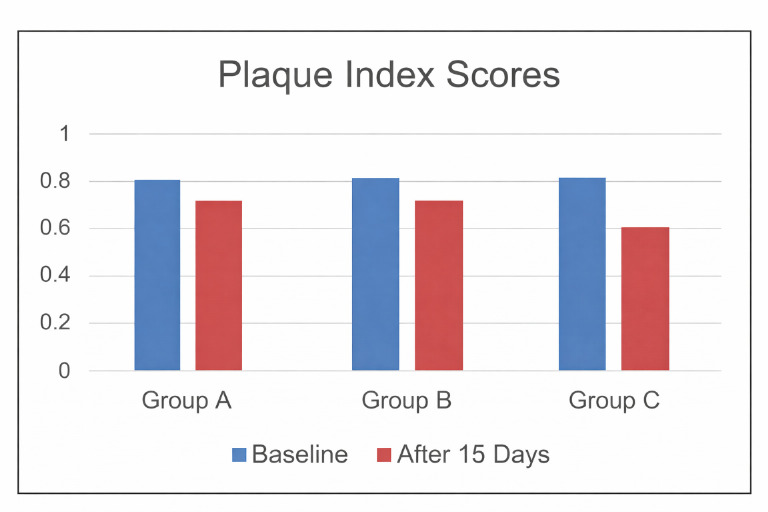
Comparison of mean plaque index scores in all three groups at baseline and after 15 days follow up period

Table 1 presents baseline and 15-day mean plaque scores (±SD) for the three groups. At baseline, values were 0.756±0.364 (Group A: Horizontal Scrub), 0.778±0.291 (Group B: Fones) and 0.805±0.382 (Group C: Modified Bass). After 15 days, reductions were noted: 0.687±0.358 (Group A), 0.680±0.267 (Group B) and 0.528±0.248 (Group C). Table 2 shows intergroup comparisons. No significant differences existed at baseline (p>0.05). At 15 days, significant differences were observed between Group C vs. A and Group C vs. B (p<0.05). Table 3 demonstrates intragroup comparisons from baseline to 15 days, revealing highly significant reductions in all groups (p=0.000). Figure 1 illustrates consistent plaque score reduction across all groups, with Group C (Modified Bass) showing the greatest improvement compared to Groups A and B.

## Discussion:

Oral health is so much connected to an individual health, the systemic illnesses are usually depicted in mouth and vice versa, low oral health can also adversely affect the overall health [1]. In developing and developing nations, oral disease has a socioeconomic effect that highlights the importance of preventive measures compared to invasive treatment [3]. The major cause of caries as well as periodontal diseases has been identified to be dental plaque as a structured biofilm; therefore, its management forms a basis in the maintenance of oral health [5]. The most feasible and economical mechanical approach of removing plaque required in the long-term oral care is tooth brushing. Despite the number of different methods of brushing, which include scrub, Bass, Stillman, Charters, Fones, selection of the best process ought to be adapted to the age, oral status and capability of the person of complying [6]. Habits of brushing teeth cannot be changed easily because motor skills should be reinforced and trained repeatedly to achieve proper brushing [7]. In this research, the three methods had a considerable effect on the reduction of plaque after 15 days and this representation demonstrates the overall advantage of brushing. Nonetheless, the Modified Bass method was superior to the Horizontal Scrub and Fones techniques, which were in line with earlier reports [8, 9]. It is effective because it is associated with improved interdental plaque removal [10, 11-12]. Being less efficient, the Fones technique is rather often suggested to young children because of its simplicity and easy mastering [6, 13 and 14]. Likewise, Horizontal Scrub technique has been regarded as appropriate to preschoolers with little motor control, but it is worse than Bass when it comes to plaque reduction [15, 16]. In general, even though brushing the teeth raises the oral health, the choice of techniques is important. The Modified Bass approach seems the best in plaque control, but simplified approaches such as Fones can be used to reinforce compliance among younger patients.

## Conclusion:

Altered Bass method was better at removing plaque, particularly in interdental and gingival regions than the Fones and horizontal scrub procedure. It is recommendable that systematic teaching of the Modified Bass technique should be incorporated into school oral health programs. Even though brushing techniques can be modified to the personal requirements, including age, motor skills and oral condition, universal instructions enhance better outcomes within the community.

## Figures and Tables

**Table 1:** Evaluation of plaque index scores in all three groups at base line and after 15days follows up period

**Variables**		**N**	**Mean**	**Std. Deviation**	**F value**	**p value**
Baseline_Plaque_score	Group A	40	0.7566	0.36472	0.19	0.82
	Group B	40	0.778	0.29102		
	Group C	40	0.805	0.38214		
Plaque_score_15_days	Group A	40	0.6873	0.35884	3.86	0.02 S
	Group B	40	0.6888	0.26786		
	Group C	40	0.5288	0.24858		
Statistical test applied:
One Way ANOVA;
S - Significant difference at p<0.05

**Table 2:** Post Hoc table

**Dependent Variable**	**(I) Group**	**(J) Group**	**Mean Difference (I-J)**	**p value**
Baseline_Plaque_score	Group A	Group B	-0.02138	0.959
		Group C	-0.04838	0.809
	Group B	Group A	0.02138	0.959
		Group C	-0.027	0.936
	Group C	Group A	0.04838	0.809
		Group B	0.027	0.936
Plaque_score_15_days	Group A	Group B	-0.0015	1
		Group C	.15850*	.047 S
	Group B	Group A	0.0015	1
		Group C	.16000*	.045 S
	Group C	Group A	-.15850*	.047 S
		Group B	-.16000*	.045 S
Statistical test applied:
Tukey test;
S - Significant at p<0.05

**Table 3:** Shows intragroup comparisons in all three brushing techniques from baseline to 15 days

		**Mean**	**N**	**Std. Deviation**	**T value**	**p value**
Pair 1	Baseline_PI_A	0.7566	40	0.36472	17.98	0.000 HS
	After_15_PI_A	0.6873	40	0.35884		
Pair 2	Baseline_PI_B	0.778	40	0.29102	5.82	0.000 HS
	After_15_PI_B	0.6888	40	0.26786		
Pair 3	Baseline_PI_C	0.805	40	0.38214	10.14	0.000 HS
	After_15_PI_C	0.5288	40	0.24858		
Statistical test applied:
Paired samples t-test;
HS - Highly significant at p<0.01
